# Miniature all-optical flexible forward-viewing photoacoustic endoscopy probe for surgical guidance

**DOI:** 10.1364/OL.400295

**Published:** 2020-11-12

**Authors:** Rehman Ansari, Edward Z. Zhang, Adrien E. Desjardins, Paul C. Beard

**Affiliations:** 1 Department of Medical Physics and Biomedical Engineering, University College London, Gower Street, WC1E 6BT, UK; 2 Wellcome/EPSRC Centre for Interventional and Surgical Sciences, University College London, 43-45 Foley Street, London W1W 7TS, UK

## Abstract

A miniature flexible photoacoustic endoscopy probe that provides high-resolution 3D images of vascular structures in the forward-viewing configuration is described. A planar Fabry–Perot ultrasound sensor with a −3dB bandwidth of 53 MHz located at the tip of the probe is interrogated via a flexible fiber bundle and a miniature optical relay system to realize an all-optical probe measuring 7.4 mm in outer diameter at the tip. This approach to photoacoustic endoscopy offers advantages over previous piezoelectric based distal-end scanning probes. These include a forward-viewing configuration in widefield photoacoustic tomography mode, finer spatial sampling (87 µm spatial sampling interval), and wider detection bandwidth (53 MHz) than has been achievable with conventional ultrasound detection technology and an all-optical passive imaging head for safe endoscopic use.

Endoscopic ultrasonography (EUS) is commonly used to assess tumors in the abdominal cavity and guide their surgical removal in minimally invasive procedures [1]. However, it provides poor label-free microvascular contrast and therefore often lacks the ability to identify deep seated tumors and delineate their boundaries based on abnormal vascular anatomy. Photoacoustic endoscopy (PAE) can potentially overcome this limitation, as it utilizes the strong optical absorption of hemoglobin to visualize tumors with high contrast [2–4]. PAE therefore offers the prospect of more sensitive tumor detection and accurate margin delineation. This could be exploited, for example, to guide the surgical excision of tumors in the liver and other abdominal organs. PAE could also improve the guidance of minimally invasive procedures in foetal medicine. For example, it could help visualize sub-surface vascular anastomoses during photocoagulation treatment of twin-to-twin transfusion syndrome [5,6]. These and other applications often require a flexible miniature photoacoustic tomography probe that provides 3D images of the vasculature in a forward-viewing configuration. However, the majority of previously reported PAE probes are sideways-viewing [7–14] and of the few forward-viewing probes demonstrated so far, almost all operate in the optical resolution microscopy mode [15,16]. In this mode, a focused excitation laser beam is raster scanned over the target tissue to generate the photoacoustic waves. This provides high optical diffraction limited lateral spatial resolution, but the imaging depth is limited to ∼1mm because the optical focus deteriorates with depth in highly scattering biological tissues.

Greater imaging depth can be achieved if the PAE probe operates in widefield tomography mode. In this approach, the target tissue is illuminated with a wide excitation laser beam and the photoacoustic waves are recorded at different spatial points resulting in acoustically defined spatial resolution. Realization of such a probe in a forward-viewing configuration requires either a highly miniaturized lateral detector scanning mechanism if a single ultrasound receiver is used or a miniature 2D detector array. However, it is challenging to fabricate a sufficiently high-density (sub-100 µm pitch) wideband (tens of MHz) ultrasound array within a millimeter-scale footprint using conventional piezoelectric detection technology [17]. Capacitive micromachined ultrasonic transducers (CMUT) arrays have been demonstrated in 1D [18], 2D [19], and ring geometries [20], and hold promise for endoscopic photoacoustic applications. However, the only previously reported forward-viewing CMUT-based PAE probe [18] employed a 1D array thus limiting the field of view to a 2D slice. Although 2D and ring CMUT array geometries can provide 3D images, they have not been demonstrated in an endoscopic PA implementation. Furthermore, as with piezoelectric detection technology, meeting the very broad bandwidth and fine spatial sampling requirements required for high resolution endoscopic PA imaging using CMUTs remains a challenge. Moreover, with a few exceptions [21,22], most piezoelectric and CMUT arrays are non-transparent. This makes it challenging to deliver a widefield excitation laser beam to the target tissue in a forward-viewing probe.

**Fig. 1.**
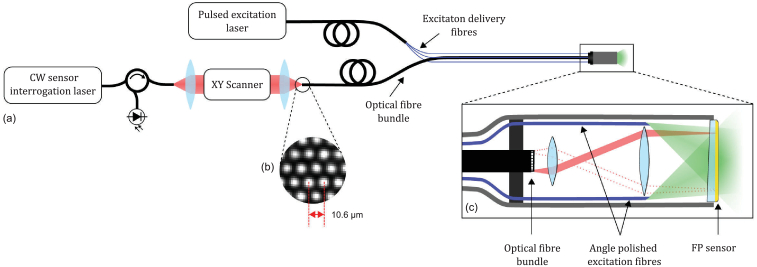
(a) Schematic of the flexible all-optical, forward-viewing PA endoscopy probe. (b) Scanned image of the fiber bundle end face (only a few cores are shown). (c) Schematic of the probe distal end that shows a planar FP sensor interrogated with fiber bundle via an optical relay, and 22° angle polished multimode fibers that deliver laser excitation light at the probe tip.

The use of a planar Fabry-Perot (FP) ultrasound sensor [23,24] offers the prospect of addressing the above challenges and realizing a miniature forward-viewing PAE probe operating in widefield tomography mode. By optically addressing the FP sensor with a coherent fiber bundle, it is possible to synthesize a very high density 2D ultrasound array. The FP sensor also offers wide bandwidth in the tens of MHz range and is transparent, thereby facilitating the delivery of the excitation laser light in a forward viewing configuration. We have previously presented a 3.2 mm diameter forward-viewing PA endoscopy probe that uses a rigid fiber bundle to interrogate an FP sensor and provides high-resolution 3D images [25]. However, the rigid nature of the probe limits the range of applications to those where line-of-sight access is available. To address this, the use of a flexible fiber bundle to interrogate the FP sensor was investigated [26]. In that study, the acoustic SNR of a fiber bundle-interrogated FP sensor was compared with a free-space FP scanning system [23] in order to identify the additional noise sources that arise due to the non-ideal light propagation characteristics of a standard commercially available fiber bundle. High quality PA images were acquired thereby demonstrating feasibility. However, it was achieved using an experimental laboratory arrangement that employed bulky cm-scale optical components and an external excitation light delivery system, with no attempt made to achieve the required level of miniaturization or integration for endoscopic use.

In this Letter, we extend the concepts described in reference [26] to realize a flexible fiber bundle based endoscopic PA probe that is sufficiently small for minimally invasive use. This required designing a miniature telecentric relay system and integrating a novel excitation light fiber delivery system within the probe tip. This resulted in a flexible probe with a 7.4 mm outer diameter tip that provides high-resolution 3D images in a forward-viewing configuration.

**Fig. 2.**
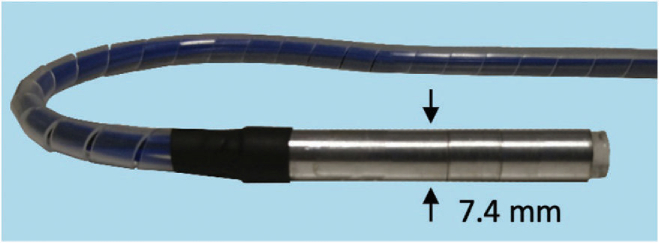
Photograph of the PAE probe distal end.

A schematic of the system is shown in Fig. 1. It comprises a flexible coherent fiber bundle, which is scanned core-by-core at its proximal end using an x–y scanner in order to address different spatial points on the FP ultrasound sensor at the probe tip. The excitation light is delivered to the probe tip through the FP sensor via an array of multimode optical fibers distributed circumferentially around the inside of the probe tip housing. The fiber bundle (Schott Inc.) comprised 18,000 fiber-optic cores with 6.7/10.26 µm core/cladding diameters, although only the central 3,000 cores were used to readout the FP sensor. Both end faces of the fiber bundle were angle polished and wedged to minimize the Fresnel reflections, which can otherwise induce noise due to parasitic interference [26]. The diameter of the bundle was 1.35 mm and protected by a flexible plastic coating with 2.2 mm diameter.

The probe tip (Fig. 1(c)) houses the FP sensor and a miniature telecentric optical relay system. The optical relay was constructed using a 6-mm diameter aspheric and achromat lenses with 3 mm and 25 mm focal lengths, respectively. The relay projects a ×8.3 magnified image of the bundle end face onto the FP sensor. This magnification was chosen to achieve a satisfactory compromise between having a large enough spot size for high acoustic sensitivity [27] and maintaining sufficiently fine spatial sampling for high spatial resolution. When the proximal end of the bundle was scanned, the relay enabled 3,000 different spatial points on the FP sensor to be individually addressed over a 6 mm diameter with a 57 µm diameter spot size and 87 µm spacing. The FP sensor was fabricated on a PMMA substrate by depositing a Parylene C spacer in between two mirror coatings. The mirror coatings were designed for high reflectivity in the spectral range 1,500–1,600 nm, where the FP sensor is interrogated, and high transmission between 580–1,250 nm to permit transmission of the excitation light through the sensor [23]. The spacer thickness was 15 µm, which provided a -3 dB acoustic detection bandwidth of 53 MHz [24]. The distal end of the bundle, relay optics, and FP sensor were housed in an aluminum tube 7.4 mm in outer diameter and 60 mm in length (Fig. 2).

**Fig. 3.**
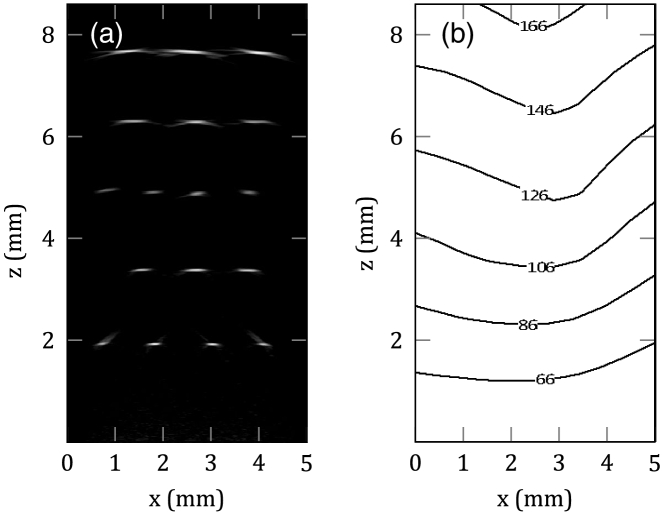
(a) x–z cross-section extracted from the reconstructed 3D PA image of a multi-layer ribbon phantom showing the cross-section of absorbing ribbons over the probe field of view. (b) A contour plot that shows the variation in the lateral spatial resolution in microns over the probe field of view.

**Fig. 4.**
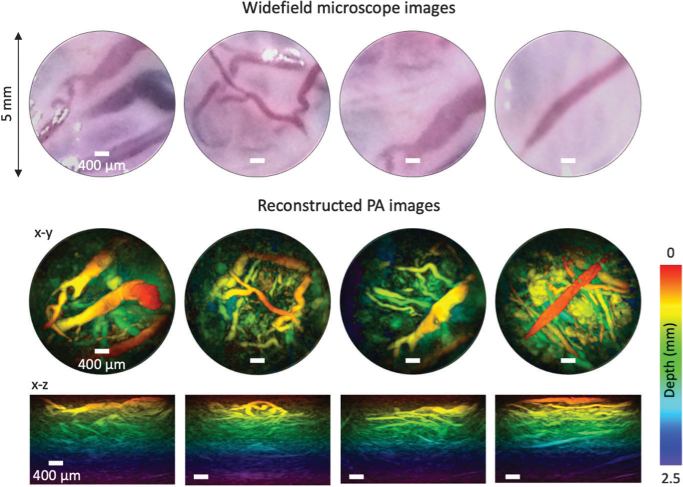
Top panel: widefield microscope images (5 mm diameter) of an *ex vivo* term normal human placenta. Middle and bottom panel: MIP PA images from the same area as top panel showing depth-resolved vascular structures. Scale bar: 400 µm; excitation wavelength: 750 nm; fluence: $4\;mJ\;{cm}^{-2}$.

The sensor was interrogated using a CW wavelength-tunable external cavity laser (1,550 nm centre wavelength, Tunics T100s-HP, Yenista Optics) in conjunction with a 100 mW EDFA (Pritel FA-23). The pulsed excitation source was a tunable (410–2,100 nm) optical parametric oscillator (OPO) based laser system (Innolas Spitlight 600), which provided 7 ns pulses at 30 Hz pulse repetition frequency (PRF). The excitation light could not be delivered through the fiber bundle, as it was not designed to deliver the required optical fluence for widefield 3D PA tomographic imaging. Thus, the output of the OPO laser was coupled into a custom made multimode fiber bundle whose distal end branches into 7 multimode fibers, with 400/440 µm core/cladding diameters. The distal ends of the fibers were polished at a 22° angle and positioned around the inner circumference of the distal end of the aluminum tube. The angle polished ends were oriented such that the light emitting from each fiber refracts towards the centre of the probe. The combined output of the 7 fibres forms a wide-field diverging laser beam 6 mm in diameter that illuminates the imaging target at the probe tip. Photoacoustic signals were recorded by optically scanning the central 3,000 cores at the proximal end of the bundle and interpolated on to a uniform rectilinear grid before reconstructing the PA images using a time reversal algorithm [28,29]. The 3D image acquisition time was approximately 100 s, which was primarily limited by the low PRF of the excitation source. However, this could be reduced to 1 s or less by using a combination of a higher PRF excitation source, a multi-beam scanning approach [30] and compressed sensing technique [31,32].

The performance of the probe was evaluated in terms of noise-equivalent pressure (NEP), field of view, and lateral and axial resolution. The mean NEP of 3,000 detection points was 600 Pa over a 20 MHz detection bandwidth. The field of view and spatial resolution was determined by imaging a multi-layer black polymer ribbon phantom that was immersed in a 1% intralipid solution. The ribbons were 20 µm thick and sufficiently wide enough that they can be regarded as approximating an absorbing step function in the x-direction. Photoacoustic signals were acquired using a 750 nm excitation wavelength and $4\;mJ\;{cm}^{-2}$ fluence. Figure 3(a) shows an x–z cross-section extracted from the reconstructed 3D PA image of the ribbon phantom, which shows individual ribbon features up to 8 mm in depth. The lateral resolution was estimated by calculating the spatial derivative of the edge-spread function obtained from the boundaries of the reconstructed features in Fig. 3(a). The contour plot in Fig. 3(b) shows the lateral spatial resolution in the x–z plane, which varies from 60 µm at a depth of 1 mm to 166 µm at a depth of 8 mm. The axial resolution of the probe was 28 µm and largely invariant over the entire depth range.

The ability of the probe to provide high-resolution 3D images in biological tissues was demonstrated by imaging an *ex vivo* human placenta. This study was approved by Joint UCL/UCLH Committees on the Ethics of Human Research (14/LO/0863). A water-based gel was used for acoustic coupling, and PA images were acquired from the chorionic side using a 750 nm excitation wavelength. The fluence at the probe tip was $4\;mJ\;{cm}^{-2}$ and thus lower than the maximum permissible exposure of $20\;mJ\;{cm}^{-2}$ for the skin [33]. The widefield microscope images from the chorionic surface of the placenta are shown in the top panel in Fig. 4. These images visualize the vasculature from the tissue surface only and are similar to those obtained by a conventional fetoscope [5]. Corresponding reconstructed 3D PA images from the same area are shown in the middle and bottom panels, color-coded according to the depth and maximum intensity projected along the orthogonal x–y and x–z planes. The PA images clearly visualize the depth resolved vascular structures of the placenta up to 2.5 mm in depth. The imaging depth is likely to have been affected by high optical absorption in dense foetal villous capillary bed, which decreases the fluence in the depth.

In summary, a novel miniature flexible PA endoscopy probe has been demonstrated that enables high-resolution 3D imaging in the forward-viewing configuration. This approach to PA endoscopy offers several advantages over the previously reported probes based on piezoelectric and CMUT technologies [7–14,16]. These include a forward-viewing configuration, finer spatial sampling, and wider detection bandwidth than achievable with piezoelectric and CMUT receivers, an all-optical passive imaging head for safe endoscopic use, immunity to electromagnetic interference (EMI), and potential compatibility with magnetic resonance (MR) scanners. Moreover, since the FP sensor is transparent in the visible and near-infrared spectral range, the probe can also integrate widefield endoscopy or other optical imaging techniques. Potential applications of the probe include delineating tumor margins in abdominal organs to guide their surgical excision, guiding needle biopsies, and assessing minimally invasive laser photocoagulation therapy in interventional procedures.
